# Factors Associated With Underprivileged E-Learning, Session Jam Phobia, and the Subsequent Mental Distress Among Students Following the Extended University Closure in Bangladesh

**DOI:** 10.3389/fpubh.2021.807474

**Published:** 2022-02-10

**Authors:** Md. Jamal Hossain, Foyez Ahmmed, Md. Moklesur Rahman Sarker, Sneha Sarwar, Md. Sazzadul Bari, Md. Robin Khan, Saimon Shahriar, Md. Oliullah Rafi, Talha Bin Emran, Saikat Mitra, Md. Rabiul Islam, Isa Naina Mohamed

**Affiliations:** ^1^Department of Pharmacy, State University of Bangladesh, Dhaka, Bangladesh; ^2^Department of Statistics, Comilla University, Cumilla, Bangladesh; ^3^Institute of Nutrition and Food Science, University of Dhaka, Dhaka, Bangladesh; ^4^Bangladesh Reference Institute for Chemical Measurements, Dhaka, Bangladesh; ^5^Department of Pharmaceutical Chemistry, Faculty of Pharmacy, University of Dhaka, Dhaka, Bangladesh; ^6^Department of Genetic Engineering and Biotechnology, Jashore University of Science and Technology, Jashore, Bangladesh; ^7^Department of Pharmacy, BGC Trust University Bangladesh, Chittagong, Bangladesh; ^8^Department of Pharmacy, Faculty of Pharmacy, University of Dhaka, Dhaka, Bangladesh; ^9^Department of Pharmacy, University of Asia Pacific, Dhaka, Bangladesh; ^10^Pharmacology Department, Medical Faculty, Universiti Kebangsaan Malaysia (The National University of Malaysia), Kuala Lumpur, Malaysia

**Keywords:** prolonged COVID-19 lockdown, underprivileged online education, severe fear of session jam, psychological distress, Bangladesh

## Abstract

Severe session jam phobia (SJP), the extent of underprivileged online education, and subsequent mental health disorders among students have emerged as distinguished global problems due to the overwhelming effects of coronavirus disease 2019 (COVID-19). The purpose of this research was to evaluate the impact of extended COVID-19 lockdown and its mediating factors on current e-Learning activities, the prevalence of severe SJP and psychological distress among university students in Bangladesh. A web-based cross-sectional study was conducted to assemble responses through Google Form by applying a simple snowball sampling technique among university students aged 18 years or above in Bangladesh. All ethical considerations were maintained, and univariate, bivariate, and multivariate analyses were employed to analyze the acquired data set. Among the total analyzed data (*n* = 1,122), the male and female ratio was almost 1:1, and a remarkable segment (63.7%) was aged between 21–24 years. Alarmingly, around 50–60% of the students were suffering from severe SJP, prevailing underprivileged education in the e-Learning platform, and severe mental distress. Logistic regression analyses demonstrated that the students from public universities, lower- and mid-income families, lower-aged, and junior years education groups were significantly (*p* < 0.05) more underprivileged than their counter groups. Besides, the monthly family income and university type significantly influenced the extent of severe SJP. Finally, the students who were female, rustic, come from low-income families (below 25,000 BDT), who had academic uncertainty, job insecurity, online exam phobia, and dissatisfaction with e-Learning education, were significantly suffering from moderate to severe mental distress. The current evidence demonstrates that a substantial number of Bangladeshi university students are struggling with extreme session jam phobia, underprivileged e-Learning education, and subsequent psychological distress, which need to be immediately addressed through concerted efforts by the government, parents, and university authorities.

## Introduction

The modern world has been crippled by the outbreak of the COVID-19 pandemic over the last 2 years, yet despite the unprecedented advancements of human knowledge and innovations, the denouement of the pandemic still seems to be far away. In this circumstance, several anti-epidemic stratagems, including lockdown and physical distancing, isolation for both infected and suspected patients, and adequate self-quarantine for traveling and other possibly exposed individuals, proved to be the exclusive and effective solutions for the containment of the pandemic (1–3). However, nationwide implementations of such drastic actions were not without repercussions as both the livelihood and lifestyles of people were heavily affected and often negatively. The sudden shift from traditional in-person classrooms to e-learning not only challenged both the teachers and students technologically but also affected the overall outcome and impacts of education at every level (4, 5).

Moreover, pandemic-associated stress, depression, and anxiety can easily undermine students' motivation for learning and derail the students from actively interacting with respective instructors through online platforms (5). Besides, the shift of the entire educational system to online platforms as forced by the pandemic is enforcing learning inadequacy among the students on the one hand while also posing considerable threats to their professional perspective on the other. Though the e-Learning recent upward trend is benefiting the developed countries due to the several advanced settings like technological merits, economic stability, well-designed infrastructures, including proper study contents, learner communities, and so on, this new online educational platform is still challenging in developing countries like Bangladesh (6). The lowered the socioeconomic status of Bangladesh attributed to the severe discrimination in modern facilities might aggravate the underprivileged e-Learning during the COVID-19 pandemic.

Bangladesh reported first the COVID-19 cases on March 8, 2020, and the government acted promptly and declared strict lockdown from March 17, 2020, enforcing complete shutdown of all government, non-government and self-governing institutions (7, 8). Although the lockdown has been loosened temporarily from time to time based on concurrent national scenarios, education institutions remained completely closed to minimize the exposure of infections and diminish the chance of any fatality among the students (9). Consequently, traditional classroom teaching became unavailable to around four million students receiving tertiary education from different public and private universities, government-accredited colleges for undergraduate, and graduate studies as well as other professional institutions in Bangladesh (10).

During this long period of the shutdown of academic institutions, e-learning methods became popular globally. However, the fundamental obstacle in efficient remote learning is the prevalence of digital inequity within the country consisting of economically challenged and unprivileged populations. Students are struggling to afford sufficiently advanced technological equipment to participate in online proceedings smoothly (11). Around 36.7% of families in Bangladesh can maintain regular access to stable internet connections, whereas only 5.6% of them possess a working computer facility (12). Eventually, technological and familial constraints experienced by many students during the pandemic are creating a distinct disparity between the educational outcomes of privileged and underprivileged classes of students (11, 13).

Moreover, financial struggles are, in turn, translating into a psychological burden on the students. A previous study delineated that the prevalence of stress, anxiety, and depression were 37.7, 40.3, and 35.2% of Bangladeshi university students, respectively (14). Eventually, the economic hardship and subsequent mental burdens generated the fear of session jam among the students both directly and indirectly. A cross-sectional study conducted in Arizona of the United States demonstrated that 13% of students were already delayed for graduation and underprivileged students were at a 55% risk of being delayed compared to their financially solvent counterparts (15).

Several factors associated with negative emotions triggered by COVID-19 lockdown have affected both the psychological and mental health of the people, especially on the students, due to the prolonged closure of the universities (16). So many studies demonstrated that almost half of the students of Bangladesh were suffering from any form of mental disorders due to this pandemic (16, 17). Another study uncovered that almost 60% of Bangladeshi university students were stressed due to the extreme academic uncertainty or delay (18). Ela et al. (12) revealed that the graduation delay is mainly occurred due to the extended closure of universities, which has rigorously augmented psychic disorders, including stress and frustration, among the university students in Bangladesh. Likewise, several reports from many other countries, such as France China and Ethiopia, illustrated that a remarkable portion of the students were having psychological problems due to COVID-19 shutdown (19–21).

Therefore, the current investigation is endeavored to identify different socioeconomic factors affecting the educational journeys of tertiary level students and evaluate such factors on how they influence the disparity between privileged and underprivileged students. Moreover, the extent of the severe session jam phobia (SJP) prevalent among the same students also needs to be ascertained to determine whether current educational disparity and depravity influence the development of such apprehension in the students. Additionally, the students affected by the prolonged university closure were also assessed to underline their afflictions from several mental health complications. Notably, the present study might bring about representative outcomes for the global community or delineate the unique impacts among university students from nations with an equivalent socioeconomic situation, no former e-Learning activities, and unprecedented extreme SJP. We also anticipate that this study results may bolster national or international lawmakers or public health experts to take appropriate initiatives in order to remove the educational uncertainty, session jam, and subsequently improve the psychological conditions of the students.

**Figure 1:**
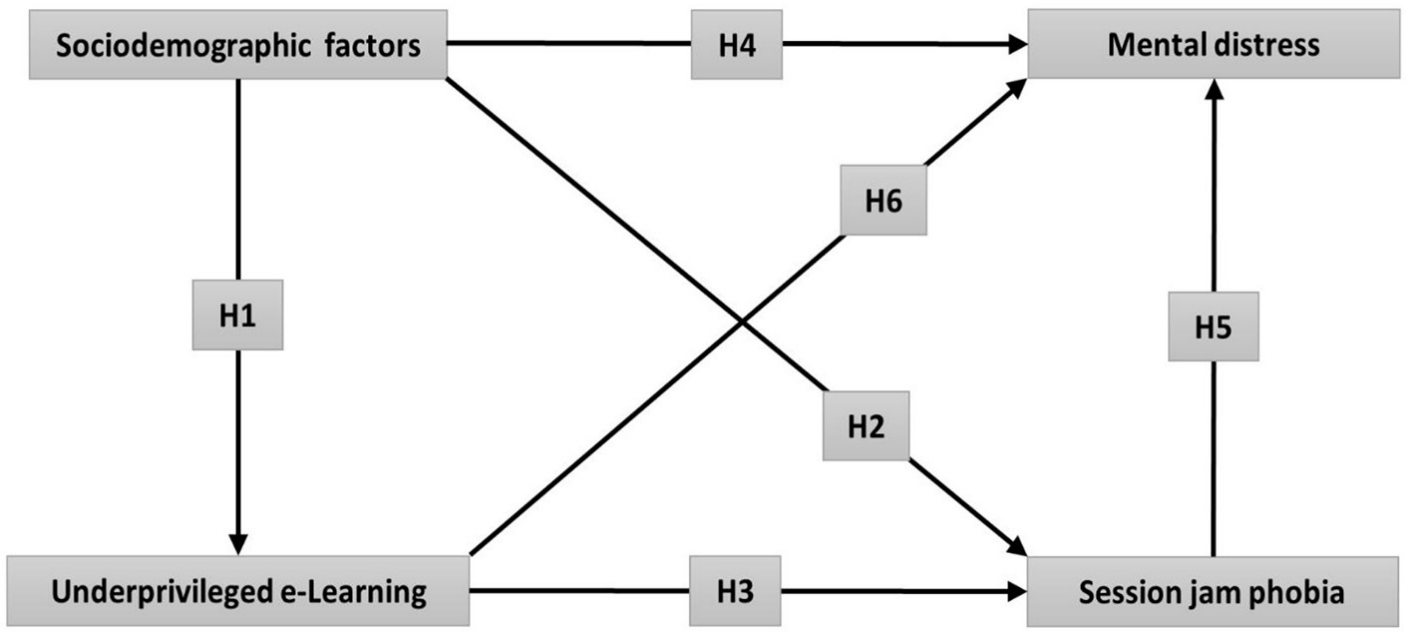
Mediating role of associated factors following prolonged COVID-19 lockdown on underprivileged online education, session jam phobia (SJP), and mental distress among university students in Bangladesh.

Moreover, several barriers and difficulties regarding the online education should urgently be solved to properly execute and drive efficiently the current e-Learning activities, alternative to the traditional class. Apart from the vulnerable e-Learning new education system, fear of academic year loss due to the prolonged COVID-19 lockdown hugely impacts severe psychological problems among university students. Therefore, the present research assessed six assembled hypotheses (Figure 1). Hypothesis 1, 2, and 4 (H1, H2, and H4): There might have significant associations between various sociodemographic factors with underprivileged e-Learning education, severe SJP, and mental distress, respectively, of university students in Bangladesh. Hypothesis 3 and 6 (H3 and H6) anticipate strong relationships between underprivileged e-Learning education with severe SJP and the mental distress of this study respondents, respectively. Finally, hypothesis 5 (H5) depicts the potential association of severe SJP with mental health distress of university students in Bangladesh.

## Methods

### Study Design

A web-based cross-sectional survey was designed with the help of Google Form, which was further segmented into four sections. Section A was intended to accumulate demographic information of the participants, including gender, age, educational level, current living area, family income, and so on. Section B included questions pertinent to unprivileged education criteria, whereas questions in Section C were assigned to evaluate the extent of SJP. Finally, section D contained the items investigating the mental distress of students due to the extended university closure due to the COVID-19 lockdown in Bangladesh. The formulated questionnaire was adapted from the previous investigations where all the queries related to the assessment of underprivileged education SJP, and mental distress were validated in the Bangladesh context (22). The questionnaire was developed in both English and Bangla versions to better understand the study details, where the forward and backward translation was executed professionally.

### Ethics

As delineated by the World Medical Declaration of Helsinki, guidelines and ethical protocols were followed and maintained strictly for this questionnaire-based research (23). Informed consent was collected from all the participants, and the data have been preserved in complete confidentiality. Ethical approval to conduct the study was secured from the Human Ethical Review Committee of State University of Bangladesh, after extensive consideration of each aspect of the study, and an official approval number (2021-7-15/SUB/H-ERC/005) was issued accordingly on July 15, 2021.

### Sampling Procedures and Participants

An online-based simple snowball sampling strategy was employed in order to assimilate the target sample into the current study (24). University students residing in Bangladesh who are aged ≥ 18 years and had minimal internet connection were presented with the study's objective and its necessity. Accordingly, following adequate understanding, those who agreed to participate in the survey were provided with the questionnaire. The questionnaire started with brief background information, its objective, and study protocol, followed by a declaration of absolute anonymity and complete privacy. Afterward, instructions on filling up the survey were detailed, and at the end, options were provided in the form of shareable links if the participants intended to share the questionnaire in their close community. As the questionnaire was circulated in prominent social media platforms, including Facebook, Messenger, Instagram, and so on, a regular user of these platforms volunteered as participants in the absence of any financial prospects. Moreover, the participants were also at complete liberty if they desired to share the questionnaire with their acquaintances, who also satisfy the inclusion criteria for the current survey. Primarily, participants were targeted from the large pool of tertiary-level students categorized under three major sections: (a) public universities, which are governed by the government, (b) private universities run by respective governing bodies, and (c) another category government-and non-government-accredited colleges for undergraduate and graduate students. The link for the Google Form was circulated through relevant groups, and pages in social media, especially Facebook, and data were collected from July 15 to August 15, 2021. At the end of this timeframe, 1,350 respondents responded to the survey. However, the total number of completed data for this study reached 1,122 for analysis after the elimination of the incomplete and partial responses.

### Measurement

#### Session Jam Phobia (SJP)

Five authorized inquiries (SJP 1–5 items: “Yes = 1” vs. “No = 0,” range: 0–5) were exercised to evaluate SJP among the Bangladeshi university students, where the cut-off points were 0–2, > 2–4, and > 4–5 for mild, moderate, and severe FSJ, respectively, according to the previously published pieces of literature (22). The Cronbach's alpha reliability coefficient value was enumerated as of 0.51 for SJP-5 items scale in this study, which indicated considerable internal consistency level of the scale (25).

#### Underprivileged E-Learning Education

The gross online access capacity below 4 h per day was the estimator to be defined as underprivileged e-Learning education at this unprecedented COVID-19 situation for Bangladesh or its equivalent socioeconomic and ethnic characteristic countries (9, 12, 26–28). One question was asked to know the total online educational hours per day of the students to measure the underprivileged e-Learning education in Bangladesh. The cut-off point was fixed for 4 h/day while defining the underprivileged e-Learning education during the COVID-19 lockdown period in this study (9, 12, 26–28).

#### Psychological Distress

In order to determine the extent and severity of the psychological distress among Bangladeshi university students, modified Kessler psychological distress scale (10-items composed of the scale; K1-10: “Yes = 1” vs. “No = 0,” Range: 0–10) was used, which was validated by previous studies (22, 29). The cut-off scores were 0–4, above 4–5, and above 5–10 for defining well, mild, moderate to severe psychologically distressed, respectively (30). The Cronbach's alpha reliability coefficient value was calculated as of 0.80 for the used Kessler psychological distress scale, which indicated an excellent internal consistency of the scale adopted in the study (25).

#### Sociodemographics

Based on several pieces of evidence and data, some relevant hypotheses might be tested to sort out a strong relationship between the current study covariates [Gender: male, female, age: ≤ 20, 21–24, > 24 years; level of schooling: lower grade-1st/2nd/3rd year, higher grade-4th/5th/Master's or higher; current living area: urban, rural; family monthly income: <25,000 BDT, 25,000–50,000 BDT, > 50,000 BDT (22, 31); university type: private, public, and others] with the outcome variables to establish the study objectives. In addition, fear of educational gap (yes, no), having job insecurity (yes, no), having online exam phobia (yes, no), and dissatisfaction toward e-Learning (yes, no) were considered as independent variables while measuring the degree of psychological distress among university students in Bangladesh.

### Data Analysis

Extensive examination of the excel datasheet, followed by subsequent exclusion of partial and incomplete data, a total of 1,122 sets of answers were available for analysis. Firstly, the data were subjected to univariate analysis through descriptive statistics (frequency and percentage distributions). Secondly, the chi-square (χ^2^) method was employed in order to ascertain various variables associated with underprivileged education, SJP, and mental distress. Thirdly, binary logistic regression was applied to evaluate the comparative susceptibility of different demographic groups with respect to one another toward the attainment of underprivileged education and the development of the severe SJP. Finally, multinomial logistic regression analysis was applied to identify significantly associated potential factors with the degree of the psychological distress of university students in Bangladesh. All the analyses were performed using IBM SPSS (version 20). The *p*-value measured at <0.05 was considered an indication of statistically significant differences being prevalent in the respective data (32).

## Results

### Demographic Characteristics

Among the total analyzed data (*n* = 1,122), the male and female ratio was almost 1:1 (male and female: 49.5 and 50.5%, respectively) (Supplementary Table 1). Besides, among the three age categories (≤ 20, 21–24, and > 24 years), 63.7% were aged between 21 and 24 years. Around 54% of the responses were higher-grade students (i.e., students of Bachelor's 4th/5th year/Master's/higher-level students). Nearly two-thirds of the students (64.8%) lived in urban areas, and more than 81% of participants came from families with monthly income below 50,000 BDT (1 USD = 84.80 BDT as of July 18, 2021). Near half of the participants spent ≥ 4 h in total for online education though only 15.3% of Bangladeshi university students had gross online access more than or equal to 6 h. Among the total students, 55.2 and 32.7% were from the private and public categorized universities, respectively. Similarly, all the demographic information was tabulated in Supplementary Table 1.

**Figure 2:**
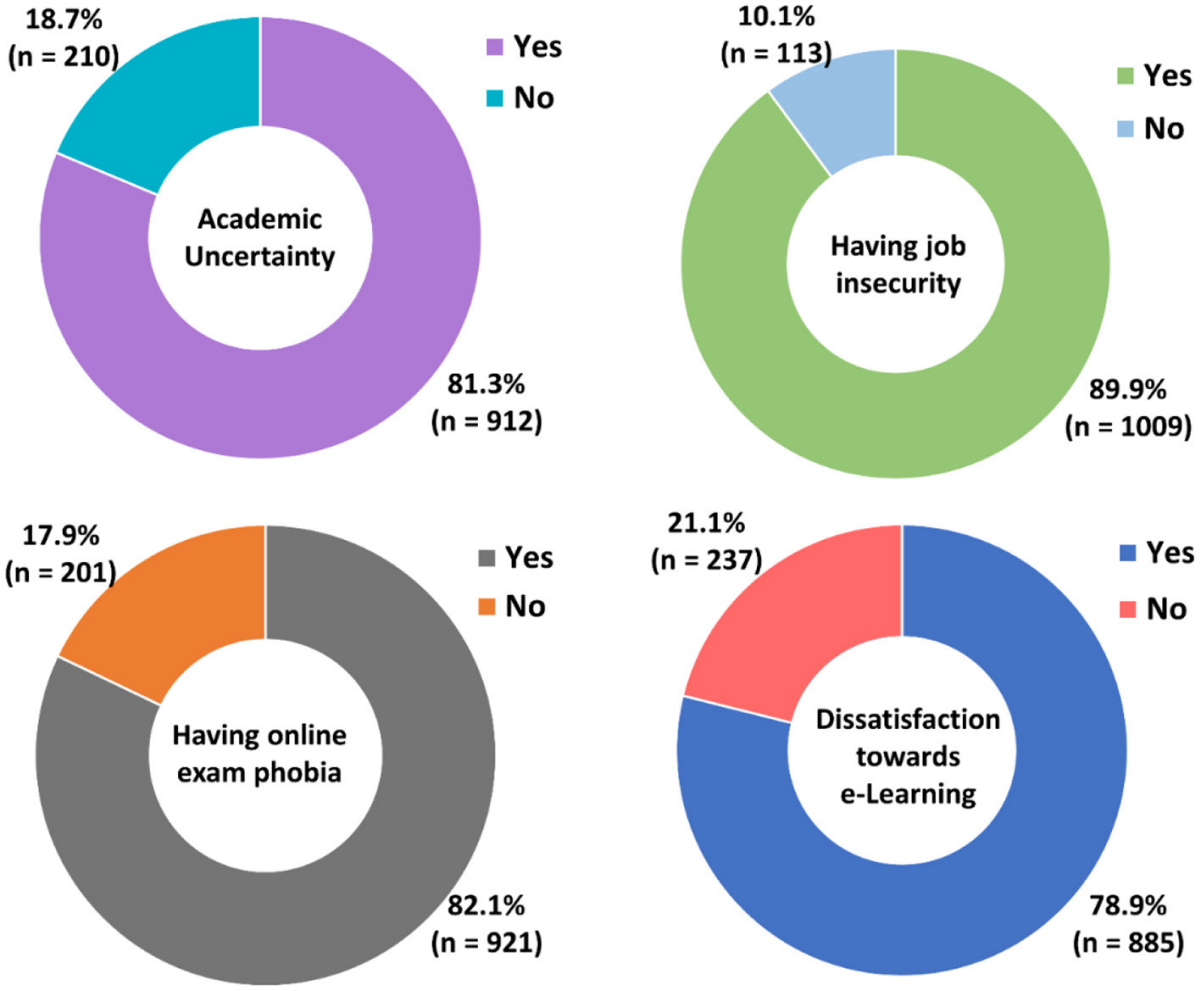
Frequency and distribution of students with academic uncertainty, job insecurity, online exam phobia, and dissatisfaction with e-Learning education following the extended university closure in Bangladesh.

**Figure 3:**
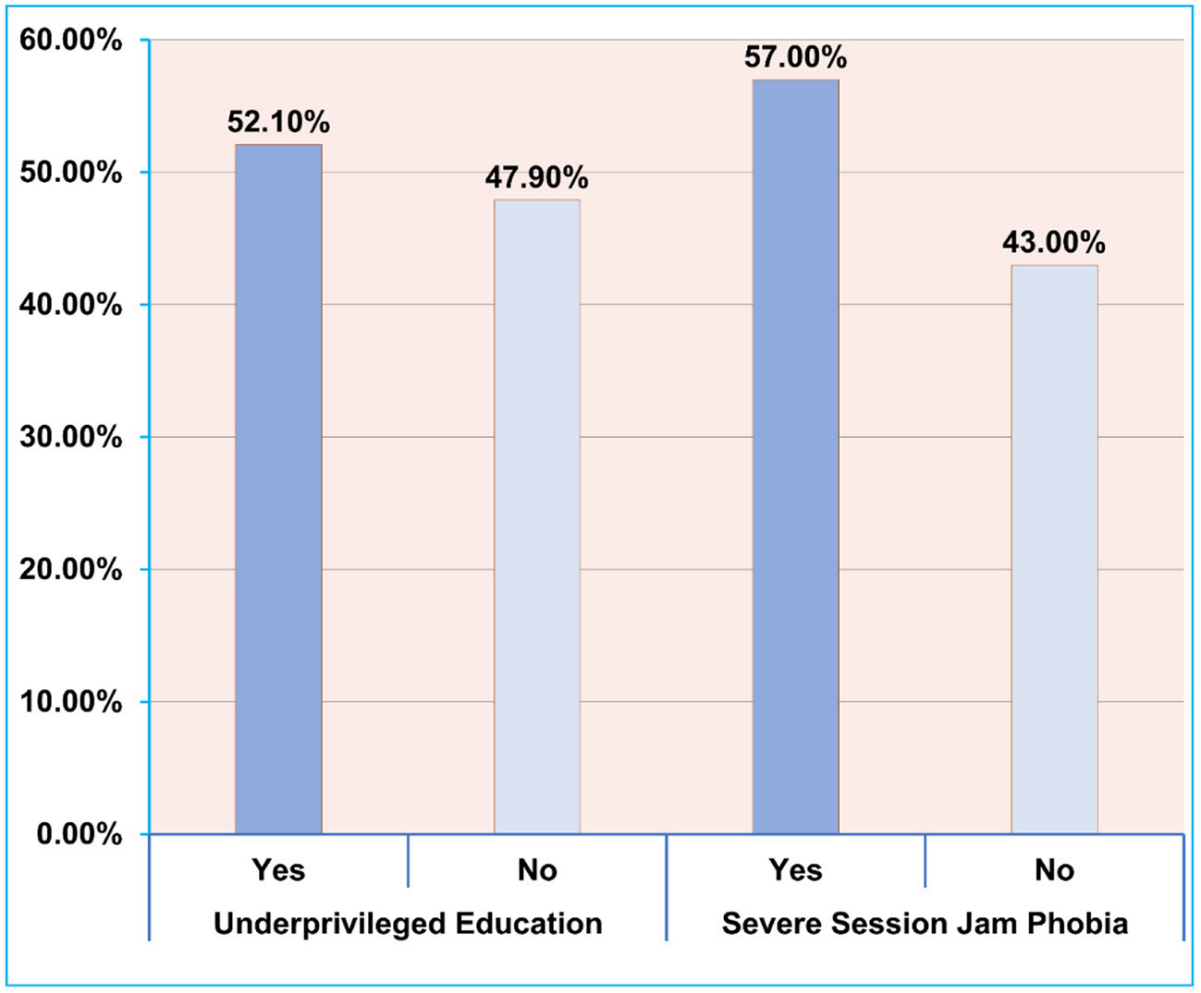
Prevalence of underprivileged online education and extreme session jam phobia (SJP) among university students following the prolonged countrywide shutdown in Bangladesh.

Moreover, more than 80% of the participants shared their opinion that they were having academic uncertainty (81.3%), future job insecurity (89.9%), and fear of online exams (82.1%) (Figure 2). Apart from this, 78.9% of the students were dissatisfied with the current e-Learning activities. Besides, nearly 60% of Bangladeshi university students were suffering from severe SJP and psychological distress. Furthermore, more than half of the participants (52.1%) reported being underprivileged from the proper online education (Figure 3).

### Chi-Square (χ^2^) Analysis of Underprivileged Education, Severe SJP, and Subsequent Mental Health Complications

While undertaking the statistical analysis, Chi-square (χ^2^) test was conducted to identify the underlying factors affecting underprivileged education, severe SJP, and subsequent mental distress. From Table 1, it has been illustrated that all the listed sociodemographic factors were significantly associated (*p* < 0.05) with underprivileged education except gender (*p* > 0.05) and residency (*p* > 0.05). There was a significant decreasing trend in the prevalence of underprivileged education with increasing age (*p* < 0.001). Alarmingly, the students from ≤ 20 years age group were 24% more underprivileged, and students from 21 to 24 years were 20.8% more underprivileged than the highest age group of students. Besides, the lower grade students were above 15% more underprivileged compared to the higher-grade students (60.1 vs. 45.0%; *p* < 0.001). Likewise, the rate of underprivileged education was a significant upward trend with decreasing family monthly income where the students from the lowest income families (<25,000 BDT) and mid-income families (25,000–50,000 BDT) were near 14 and 10% more underprivileged, respectively, than the students from the highest income families (>50,000 BDT). Besides, the university category significantly influenced the prevalence of underprivileged rate to current online education (*p* = 0.002).

**Table 1:** Chi-square (χ^2^)-test for evaluating the association of different factors with underprivileged education and severe session jam phobia (SJP) among university students following extended COVID-19 shutdown in Bangladesh.

**Covariates**	**Options**	**Underprivileged e-Learning education**								**Severe session jam phobia (SJP)**							
		**No**		**Yes**		***df***	**χ^2^-value**	***P*-value**	**Cramer's V**	**No**		**Yes**		***df***	**χ^2^-value**	***P*-value**	**Cramer's V**
		***N***	**%**	***N***	**%**					***N***	**%**	***N***	**%**				
Gender	Male	257	46.3	298	53.7	1	1.189	0.276	0.032	242	43.6	313	56.4	1	0.138	0.710	0.011
	Female	281	49.6	286	50.4					241	42.5	326	57.5				
Age (years)	≤ 20	58	40.3	86	59.7	2	**37.105**	**< 0.001**	0.181	52	36.1	92	63.9	2	**10.042**	**0.007**	0.094
	21–24	311	43.5	404	56.5					333	46.6	382	53.4				
	> 24	169	64.3	94	35.7					98	37.3	165	62.7				
Education level	Lower grade	209	39.9	315	60.1	1	**25.62**	**< 0.001**	0.151	217	41.4	307	58.6	1	1.073	0.300	0.030
	Higher grade	329	55.0	269	45.0					266	44.5	332	55.5				
Current living area	Urban	350	48.1	377	51.9	1	0.030	0.861	0.005	334	45.9	393	54.1	1	**7.055**	**0.008**	0.079
	Rural	188	47.6	207	52.4					149	37.7	246	62.3				
Monthly family income (BDT)	<25,000	165	37.8	271	62.2	2	**34.526**	**< 0.001**	0.175	159	36.5	277	63.5	2	**24.724**	**< 0.001**	0.148
	25,000–50,000	246	51.5	232	48.5					205	42.9	273	57.1				
	> 50,000	127	61.1	81	38.9					119	57.2	89	42.8				
University category	Private	321	51.9	298	48.1	2	**12.755**	**0.002**	0.106	333	53.8	286	46.2	2	**65.583**	**< 0.001**	0.242
	Public	148	40.3	219	59.7					113	30.8	254	69.2				
	Others	69	50.7	67	49.3					37	27.2	99	72.8				
Underprivileged e-Learning	Yes									254	43.5	330	56.5	1	0.098	0.754	0.009
	No									229	42.6	309	57.4				

*“Private” and “public” universities are private sector and government owned universities, respectively. “Others” category represents both government and private owned equivalent institutions. BDT = Bangladeshi taka; 1 USD = 85.56 BDT as of October 27, 2021. Lower grade = 1st/2nd/3rd year level students, and higher-grade students = 4th/5th/Master's or higher-level students. df, degree of freedom*.

*The bold values indicate the statistically significant results*.

Most of the considered sociodemographic factors were significantly associated with extreme SJP except few elements in the χ^2^-test. Notably, the students from rural areas suffered from 8.2% more severe SJP than the students from urban areas (62.3 vs. 54.1%; *p* = 0.008). Besides, the students from the lowest-income and mid-income families were 20.7 and 14.3% more feared from session jam than those from the highest-income families (*p* < 0.001). The students from other categories and public universities were suffering from tremendously higher severe SJP (fear difference = 26.2 and 23%, respectively; *p* < 0.001) than the private university students.

Moreover, Table 2 revealed that all the covariates, except underprivileged e-Learning education, were significantly associated with several degrees of mental distress. Respondents from the lowest age group (≤ 20 years) were most affected by moderate to severe distress compared to higher-aged group students. The higher-grade students were 7.5% more likely to be mild to moderately distressed than the lower-grade students (72.1 vs. 64.6%; *p* = 0.015). Besides, students from rural areas were 13.3% more likely to be moderate to severely stressed than those from urban areas (76.7 vs. 63.4%; *p* < 0.001). A higher percentage of students from lowest- and medium-income families were suffering from moderate to severe distress than high-income families (77.3, 65.5%, respectively, vs. 54.8%; *p* < 0.001). Besides, the respondents from public universities exerted significantly more distress than their counter groups. Similarly, students with academic uncertainty, job insecurity, online exam phobia, and dissatisfaction with current e-Learning activities were significantly suffering from more moderate to severe psychological distress than their peer groups. Finally, the students who had severe SJP were suffering from 45.7% more moderate to severe distress than those who had no severe SJP.

**Table 2:** Chi-square (χ^2^) analysis for evaluating the association of different cofactors with several degree of mental distressed conditions among students following extended university closure in Bangladesh.

**Covariates**	**Options**	**Not distressed**		**Mild distressed**		**Moderate to Severely distressed**		***df***	**χ^2^-value**	***P*-value**	**Cramer's V**
		***N***	**%**	***N***	**%**	***N***	**%**				
Gender	Male	136	24.5	59	10.6	360	64.9	2	**5.442**	**0.07**	0.069
	Female	117	20.6	46	8.1	404	71.3				
Age (years)	≤ 20	18	12.5	12	8.3	114	79.1	4	**13.837**	**0.008**	0.078
	21–24	173	24.2	75	10.5	467	65.3				
	> 24	62	23.6	18	6.8	183	69.6				
Education level	Lower grade	99	18.9	47	9.0	378	72.1	2	**8.348**	**0.015**	0.086
	Higher grade	154	25.8	58	9.7	386	64.6				
Current living area	Urban	187	25.7	79	10.9	461	63.4	2	**20.887**	**< 0.001**	0.136
	Rural	66	16.7	26	6.6	303	76.7				
Monthly family income (BDT)	<25,000	68	15.8	30	6.9	337	77.3	4	**36.173**	**< 0.001**	0.126
	25,000–50,000	118	24.7	47	9.8	313	65.5				
	> 50,000	66	31.7	28	13.5	114	54.8				
University type	Private	177	28.6	55	8.9	387	62.5	4	**31.784**	**< 0.001**	0.119
	Public	54	14.7	32	8.7	281	76.5				
	Others	22	16.2	18	13.2	96	70.6				
Fear of educational gap	Yes	151	16.6	75	8.2	686	75.2	2	**120.63**	**< 0.001**	0.327
	No	102	48.6	30	14.3	78	37.2				
Having job insecurity	Yes	193	19.1	85	8.4	731	72.4	2	**89.253**	**< 0.001**	0.282
	No	60	53.1	20	17.7	33	29.2				
Having online exam phobia	Yes	155	16.8	78	8.5	688	74.7	2	**122.75**	**< 0.001**	0.330
	No	98	48.8	27	13.4	68	37.9				
Dissatisfaction toward e-Learning	Yes	148	16.7	71	8.0	666	75.2	2	**102.61**	**< 0.001**	0.302
	No	105	44.3	34	14.3	98	41.3				
Underprivileged e-Learning	Yes	129	22.1	49	9.1	365	67.8	2	0.924	0.910	0.028
	No	124	23.0	56	9.6	399	68.3				
Severe Session jam phobia	Yes	118	13.9	59	6.9	673	79.2	2	**201.94**	**< 0.001**	0.424
	No	135	49.8	46	16.9	91	33.5				

*The bold values indicate the statistically significant results*.

### Binary Logistic Regression Analysis of Underprivileged E-Learning and Severe SJP

Binary logistic regression was employed during the multivariate analysis to investigate the significant association of several sociodemographic factors with underprivileged online education and severe FSJ and the findings were summarized in Table 3. The students from ≤ 20 and 21–24 years age groups were 2.27 times (95% confidence interval [CI] = 1.35, 3.81; *p* = 0.002) and 2.34 times (95% CI = 1.655, 3.306; *p* < 0.001) more likely to be underprivileged from the current online education than the students from > 24 years age group. Similarly, in reference to high income families, the risk of underprivileged education was significantly higher for the students from lower and middle-income families [adjusted odds ratio (AOR) = 2.378, 95% CI = 1.647, 3.434; *p* < 0.001 and AOR = 1.431, 95% CI = 1.014, 2.020; *p* = 0.041, respectively]. Besides, public university students were 1.65 times more likely to be underprivileged than the private university students in this current online education platform (AOR = 1.655, 95% CI = 1.245, 2.201; *p* = 0.001).

**Table 3:** Logistic regression analysis for finding potential factors associated with underprivileged e-Learning education and severe session jam phobia (SJP) among university students following prolonged COVID-19 lockdown in Bangladesh.

**Covariates**	**Options**	**Underprivileged e-Learning education**				**Severe session jam phobia (SJP)**			
		**AOR**	***P*-value**	**95% CI**		**AOR**	***P*-value**	**95% CI**	
Gender	Male vs. Female^R^	1.198	0.166	0.928	1.547	0.825	0.142	0.638	1.066
Age (years)	≤ 20 vs. > 24^R^	2.269	**0.002**	1.350	3.813	1.012	0.965	0.596	1.717
	21-24 vs. >24^R^	2.339	**< 0.001**	1.655	3.306	0.766	0.131	0.542	1.083
Education level	Lower grade vs. higher grade^R^	1.313	**0.074**	0.974	1.768	1.086	0.589	0.805	1.465
Current living area	Urban vs. Rural^R^	1.291	0.071	0.979	1.703	1.003	0.981	0.760	1.325
Monthly family income (BDT)	<25,000 vs. > 50,000^R^	2.378	**< 0.001**	1.647	3.434	2.079	**< 0.001**	1.438	3.006
	25,000–50,000 vs. > 50,000^R^	1.431	**0.041**	1.014	2.020	1.688	**0.003**	1.198	2.378
University type	Public vs. private^R^	1.655	**0.001**	1.245	2.201	2.390	**< 0.001**	1.794	3.184
	Others vs. private^R^	1.185	0.412	0.790	1.778	2.732	**< 0.001**	1.776	4.204
Underprivileged e-Learning	Yes vs. No^R^					0.836	0.170	0.647	1.080
Constant		0.198	**< 0.001**			0.746	0.222		

*R, reference group*.

*The bold values indicate the statistically significant results*.

Moreover, the risk of severe SJP was two times and 1.69 times higher for the students from lower-income (95% CI = 1.438, 3.006; *p* < 0.001) and middle-income families (95% CI = 1.198, 2.378; *p* < 0.003) compared to the students from high-income families. Notably, the AOR of severe SJP was around 2.4 and 2.74 times higher among students of public universities (95% CI = 1.794, 3.184; *p* < 0.001) and other institutions (95% CI = 1.776, 4.204; *p* < 0.001), respectively, compared to the private university students. The students having online education for ≥ 6 h, students who were spending 4–<6 h (AOR = 1.522, 95% CI = 1.035, 2.237; *p* = 0.033) were having a higher risk of severe SJP. Likewise, all the potential variables associated with underprivileged e-Learning online education and severe SJP were included in Table 3 with their odds ratio upon adjusting other factors.

### Multinomial Logistic Regression Analysis of Degree of Psychological Distress

According to Table 4, the probability of male participants of being moderate to severely stressed was significantly 31% lower than females (AOR = 0.695; 95% CI = 0.492, 0.982; *p* = 0.039). The urban students were having 40% less likely to be moderate to severely stressed compared to the rustic students (AOR = 0.602; 95% CI = 0.86, 1.92; *p* = 0.009). Students whose family income was below 25,000 BDT were 1.7 times more at risk of experiencing moderate to severe stress than students with family income above 50,000 BDT (95% CI = 1.092, 2.850; *p* = 0.020). Besides, the participants with academic uncertainty, job insecurity, online exam phobia, and dissatisfaction with current online education were more prone to moderate to severe psychological distress than their reference groups (AOR = 2.2–2.6; *p* < 0.001). Similarly, all the potential cofactors associated with mild or moderate to severe psychological distress were summarized in Table 4.

**Table 4:** Multinomial logistic regression analysis for finding the association of cofactors with several psychological distress conditions among the students following extended closure of university in Bangladesh.

**Covariates**	**Options**	**Mild distressed**				**Moderate to severely distressed**			
		**AOR**	**95% CI**		***P*-value**	**AOR**	**95% CI**		***P*-value**
			**Lower**	**Upper**			**Lower**	**Upper**	
Gender	Male vs. Female ^R^	1.167	0.708	1.922	0.545	0.695	0.492	0.982	**0.039**
Age (years)	≤ 20 vs. >24 ^R^	2.396	0.825	6.962	0.108	1.745	0.821	3.710	0.148
	21–24 vs. >24 ^R^	1.765	0.911	3.419	**0.092**	0.873	0.562	1.356	0.546
Education level	Lower grade vs. higher grade ^R^	0.942	0.541	1.638	0.832	1.196	0.811	1.764	0.367
Current living area	Urban vs. Rural ^R^	1.282	0.725	2.267	0.392	0.602	0.412	0.881	**0.009**
Family income (BDT)	<25,000 vs. > 50,000 ^R^	0.842	0.430	1.651	0.618	1.764	1.092	2.850	**0.020**
	25,000–50,000 vs. > 50,000 ^R^	0.854	0.477	1.529	0.595	1.162	0.756	1.784	0.493
University type	Private vs. others ^R^	0.374	0.178	0.785	**0.009**	1.029	0.577	1.832	0.924
	Public vs. others ^R^	0.718	0.323	1.595	0.416	1.644	0.886	3.050	0.115
Academic uncertainty	Yes vs. No ^R^	1.820	0.911	3.635	**0.090**	2.471	1.479	4.128	**0.001**
Having job insecurity	Yes vs. No ^R^	1.308	0.655	2.609	0.447	2.413	1.356	4.295	**0.003**
Having online exam phobia	Yes vs. No ^R^	1.881	0.982	3.601	**0.057**	2.215	1.379	3.558	**0.001**
Dissatisfaction toward e-Learning	Yes vs. No ^R^	1.405	0.782	2.526	0.256	2.595	1.687	3.992	**< 0.001**
Underprivileged e-Learning	Yes vs. No ^R^	1.046	0.645	1.696	0.855	0.985	0.702	1.380	0.928
Severe SJP	Yes vs. No ^R^	0.531	0.224	1.262	0.152	1.485	0.800	2.756	0.210
Constant		0.210			**0.006**	0.130			**< 0.000**

*R, reference group*.

*The bold values indicate the statistically significant results*.

## Discussion

As the education system is undeniably affected by the COVID-19 pandemic, the exact aim of the study has been to identify the association of various socioeconomic factors and their effects on underprivileged education, extreme SJP, and subsequent mental health complications. Additionally, the study has endeavored to integrate these three outcome variables. It is evident from the current study that more than half of the university students in Bangladesh are currently suffering from extreme fear of session jam, underprivileged online education, and severe psychological distress due to prolonged COVID-19 shutdown. Overall, the study tested six hypotheses illustrated in Figure 1, where we found that the mediating sociodemographic factors impacted underprivileged e-Learning, severe SJP, and subsequent mental distress of the university students in Bangladesh.

Bangladesh is an economically challenged country with around 13% of the students lack the availability of an appropriate electronic device like a smartphone (33). It is mandatory to have at least a smartphone along with a stable internet connection; in contrast, attendance of online classes regularly and efficiently is often disrupted. Bangladesh Bureau of Statistics reported that 62% of the households in Bangladesh do not have stable internet at home, and only 8.7% of the poorest households have internet (34). Besides, near 50% of students from various public universities in Bangladesh failed to bear internet charges during the COVID-19 crisis (35). The aforementioned reasons could clarify why about 52.1% of our participants were away from appropriate e-Learning. Moreover, along with the reduction in active study hours, a general decrease in attendance has also been observed by an earlier study as the overall attendance in online classes reached as low as 40% (36). Another investigation reported a significant decrease in study time by more than 2 h amid the COVID-19 lockdown (35).

In the current study, ~6 in every 10 participants expressed their fear of session jam. In this context, earlier studies suggested that the inadequate experience with online academic proceedings along with the difficulties presented by the online enrollment procedures have instilled confusion and lack of confidence among the students. A majority of these students (around 60%) are in turn refraining from participating actively during online classes and experiencing fear of academic gap on an unprecedented scale (22, 35). A national report has recently stated that four million university students are currently seized by the fear of session jam, and 2.6 million are completely away from proper e-Learning activities in Bangladesh (37). This fear, in turn, has mutated into psychological stress in many of the affected students (16, 38, 39). According to a recent study, around 80% of the students having extreme fear of academic uncertainty were experiencing critical mental distress (18). In another study, Shafiq et al. (40) reported that 77% of the students from public universities in Bangladesh were mentally distressed because of the economic crisis and emerging session jam, whereas around 80% of private university students were psychologically disturbed because of uncertainties of online activities and expensive education expenses.

### Underprivileged E-Learning Education and Its Associated Factors (H1)

Findings of the current study enumerated age, financial condition, university types as the significant independent factors associated with underprivileged online education. Near 60% of the students aged up to 24 years were experiencing underprivileged education. The income of almost 36 million people was downsized during the current COVID-19 lockdown, leading to a significant financial crisis in additional 16 million families along with the already economically challenged families of the country (41). As young learners are primarily dependent on their families for their education, any obstacle in the family income also affects their education. Hence, the current data endorsed that the more youthful students were almost twice higher risk of underprivileged education than the older ones.

Moreover, around 62.1% of the students from low-income families were more prone to underprivileged education than students from high-income families. This problem may have stemmed from their inability to access improved electronic devices and strong internet connections (42). According to a previous study, 50% of the absent students could not attend online classes due to device availability (35). Furthermore, early literature suggested that apart from the students' limited access to these tools, inappropriate home environments may have also turned the education system unfavorable (43). Public university students were found to be 65% more at risk of underprivileged education than private university students. Around 300,000 students are studying in the 44 public universities of Bangladesh (37), most of whom come from unprivileged socioeconomic backgrounds and have to bear their educational expenses by their own incomes (40). However, about 75% of such students lost their income completely during the pandemic and are suffering from an unfathomed economic crisis (35). Furthermore, a majority of the public universities in Bangladesh lacked adequate facilities to conduct successful online classes by themselves, and such students have also declined the hope for institutional support that has been necessary for their studies (44). Eventually, the students from public universities suffered academically to a greater extent than private university students and experienced prominent underprivileged education. The findings of the limited exposure of public university students to online education have been parallel to previous literature (40). As most of the resident students of public universities are from small town, or rural areas (40), poor internet connection, limited electricity, and high internet costs have limited their access to the current e-Learning platform (45). Therefore, hypothesis 1 (H1) has been confirmed where several sociodemographic factors significantly influence the underprivileged e-learning education during the COVID-19 lockdown in Bangladesh.

### Severe SJP and Its Associated Factors (H2 and H3)

Due to the current pandemic situation, the educational system of Bangladesh has taken a different and drastic turn to the online education platform. Nevertheless, students of different socioeconomic and demographic classes face some unavoidable barriers that narrow the efficiency of the current online classes and pose additional concerns to the students. Accordingly, monthly family income and university type were found to be significantly associated with severe SJP prevalent among the students in our modeling study. Though age group significantly influenced the prevalence of severe SJP in the χ^2^-test, the factor was not found to be a significant association in regression analysis. Around 60% of the students from low-income and middle-income families expressed extreme SJP, and these two groups of students were about 2 times and 1.7 times higher risk, respectively, of having severe SJP compared to the students of high-income families. The unemployment rate is rising sharply in Bangladesh during this COVID-19 pandemic (45). To cope with this sudden change in income, families are restricting their budgets in different sectors, and presumably, greater expenses on internet subscriptions and buying better smartphones are not often prioritized (40, 46). According to a previous finding, 30% of the students needed subsidies or additional economic support from outside the family to afford a smartphone, and 60% of the students from public universities needed subsidies to maintain monthly internet subscriptions (35). Subsequently, students from low-income families are lagging in online classes and having SJP to greater extents.

Moreover, most public universities have been progressing slowly in terms of online classes, which is delaying the regular session of the students. Technological limitations, in turn, may undermine the efficiency of the classes and thereby imposes greater SJP among students. Students of public universities and from low-income families were found to be the prime victims of such scenarios. As reported earlier, 76% of private university students and only 30% of public university students can attend online classes, while others cannot, primarily due to slow internet connectivity and financial crises (40). Furthermore, the areas of residency of the students and their financial conditions have made them a victim of underprivileged education. Public universities typically have a larger number of students than private universities and bringing all of them in one single virtual platform often appears to be complicated. Moreover, public universities in Bangladesh have rarely possessed updated technological facilities. Eventually, a large number of public universities, due to their lack of technical resources, could not initiate online classes timely and could not conduct any class assessment, accordingly, forcing them to lag (40). Therefore, institutions' shortcomings on one hand and students' limitations on the other, as enforced by this updated and expensive educational system, have created a greater fear of session jam. However, the current data traced no significant association of students' underprivileged status with their SJP as around 60% of privileged and underprivileged groups had severe SJP. This outcome may have stemmed from the fact that regardless of their privilege status, most of them were experiencing similar fear of academic delay due to late initiation of online classes, disrupted class schedules, lack of understanding over the subject matter, and repeated delay in examinations. So, it can be noted that our findings confirmed hypothesis 2 (H2), but hypothesis 3 (H3) has not been established in this study settings.

### Psychological Distress and Its Associated Factors (H3, H4, and H5)

Students' mental health issues are being exacerbated by the lengthy confinement at home and academic and career uncertainty during the pandemic time (47–49). In light of the current circumstances, the study has underlined how e-Learning activities, fear of academic gap, online examination phobia, and academic uncertainty, including several sociodemographic factors, has sparked psychological distortions of Bangladeshi university students during the extended university closure.

The current analysis revealed that the female students were experiencing more stress than males. This outcome is supportive of several previous reports (50–52). One reason could be that women or female students are more likely to do extra household chores during emergencies, putting them at a higher risk of being vulnerable (14). Contrary, some studies have found no significant differences in depression or anxiety between male and female students (24, 53, 54). Our research also demonstrated that students from urban regions were 44% less distressed than pupils of rural areas. This could be attributed to rural students' low socioeconomic status and unsatisfactory internet connections preventing them from attending online classes (55). Additionally, the lowest-income family students were 1.76 times higher stressed than the students of the highest-income families, that was consistent to the previous findings reported by Sayeed et al. (56). The underlying reason for extreme stress among low-income family students might be that they are incapable of participating in the present e-learning programs and are concerned about their academic gap and job security.

To the best of our knowledge, this is the first study in Bangladesh, where we have investigated the potential impacts of current academic uncertainty, job insecurity, online exam phobia, dissatisfaction toward e-Learning education on the psychological stress of the students. The students with academic uncertainty and job insecurity were nearly 2.5 times more likely to be moderate to severely distressed than their reference group. It is comprehensible that COVID-19 induced academic uncertainty has exacerbated job insecurity among university students, triggering psychological depression, and anxiety (57). Besides, according to our outcomes, the online exam phobia and dissatisfaction with the current online educational programs significantly influenced the mental health complications. The rapid shift to virtual teaching and students' dissatisfaction with online education have contributed to increased anxiety and stress levels. This might be attributed to a lack of internet connectivity and their instructors' overbearing demands, which include several assignments and strict deadlines (58, 59). Additionally, students' lack of experience with information technology causes it more challenging to adapt to the e-learning environment, resulting in high academic stress (60). Though hypothesis 4 (H4) has been proved established, hypotheses 5 and 6 (H5 and H6) were not confirmed in the study because we did not find a significant association of underprivileged e-Learning and severe session jam phobia with the students' mental distress.

## Practical Implications

According to our searching experience, this is the first study for obtaining the significantly associated factors with underprivileged education, extreme SJP, and subsequent mental complications among Bangladeshi university students following prolonged COVID-19 lockdown. The conclusions attained in this report might interpret the prevailing fear of session jam, underprivileged online education and subsequent psychological stress for university students of other countries having equivalent socioeconomic and educational backgrounds to Bangladesh. The study outcomes will further promote the institutional administrations, educationalists, and the government to identify the disastrous condition of the ongoing online academic status and SJP, including the psychological well-being of the current university students, and take more adamant stratagems to immediately fix these integrated problems. Besides, the epidemiologists will be able to introduce proper interventions to lessen the severe SJP and subsequently fasten the process of a re-initiating educational institution to decrease the anticipated academic vulnerability and negative psychological impacts.

## Limitations

The major limitation is that the study was entirely based on the students' opinions; intrinsic bias may have been introduced by the unruly, undisciplined, and irregular students, who are always finding excuses to avoid any educational activities. However, the disparities in both the internet service and internet access across the country have obstructed such opportunities and may require extensive offline, personal interviews to acquire a better realization of the current conditions, which also seems impractical during the pandemic. Furthermore, the current research did not cover all the potentially crucial parameters of the existing educational platform, which might have a likely association with severe SJP. Nevertheless, we found no significant influence of underprivileged education on severe SJP and psychological distress, which justifies further persuasive evaluation to be determined for the relationship of the rest of the factors with SJP and mental health complications among university students during the prolonged COVID-19 lockdown in Bangladesh.

## Conclusions

The global COVID-19 pandemic is taking a tremendous toll on people of all aspects of life, and students are also becoming a victim of this unavoidable situation. The prolonged closure of educational institutions and shifting from traditional to online learning is worsening the situation for students. Our findings demonstrated that an unusual share of university students have underprivileged access to current online education, severe SJP, and subsequently been suffering from moderate to severely psychological disorders. The study identified several socioeconomic indicators that are influencing these scenarios. Several factors, including the age of the students, their residency, the type of institution, were significantly contributing to this underprivileged education and severe SJP. Furthermore, apart from several sociodemographic factors, online exam phobia, academic uncertainty, job insecurity, and dissatisfaction with e-Learning activities have severely disrupted the students' mental health. These profound problems must be immediately addressed through a combination effort by the government, university authorities, and parents.

## Data Availability Statement

The original contributions presented in the study are included in the article/Supplementary Material, further inquiries can be directed to the corresponding author/s.

## Ethics Statement

Informed virtual consent was collected from all the participants, and the data have been preserved in complete confidentiality. Ethical approval to conduct the study was secured from the Human Ethical Review Committee of State University of Bangladesh, after extensive consideration of each aspect of the study, and an official approval number (2021-7-15/SUB/H-ERC/005) was issued accordingly on July 15, 2021.

## Author Contributions

MJH developed the idea of the work. MJH and FA designed the study. MJH, FA, MMRS, MOR, TBE, SM, and MRI collected the data. MJH, FA, and SSa cured and analyzed the raw data. MJH and FA interpreted the analyzed data. MJH, SSa, MSB, MRK, and SSh searched the literature and drafted the original manuscript. MJH, MMRS, and INM have made funding acquisitions. MJH, MMRS, MOR, TBE, SM, MRI, and INM critically revised and improved the manuscript. All authors reviewed and approved the final version of the manuscript.

## Conflict of Interest

The authors declare that the research was conducted in the absence of any commercial or financial relationships that could be construed as a potential conflict of interest.

## Publisher's Note

All claims expressed in this article are solely those of the authors and do not necessarily represent those of their affiliated organizations, or those of the publisher, the editors and the reviewers. Any product that may be evaluated in this article, or claim that may be made by its manufacturer, is not guaranteed or endorsed by the publisher.
