# Supplementary material for: Factors Associated With Underprivileged E-Learning, Session Jam Phobia, and the Subsequent Mental Distress Among Students Following the Extended University Closure in Bangladesh
Source: Front Public Health. 2022 Feb 10;9:807474. doi: 10.3389/fpubh.2021.807474 (PMC8868938; doi:10.3389/fpubh.2021.807474)
Supplement: Supplementary file 1 [file Table_1.DOCX]

Supplementary Material

**Factors associated with underprivileged e-Learning, session jam phobia, and the subsequent mental distress among students following the extended university closure in Bangladesh**

**Table S1** Descriptive statistics (frequency and percentage distributions) of demographic characteristics and covariates to investigate severe session jam phobia, underprivileged e-Learning education, and the degree of psychological distress conditions among the university students following the extended COVID-19 lockdown in Bangladesh.

| **Variables** | **Options** | **Number (N)** | **Percentage (%)** |
| --- | --- | --- | --- |
| **1. Gender** | Male | 555 | 49.5 |
|  | Female | 567 | 50.5 |
| **2. Age (years)** | ≤ 20 | 144 | 12.8 |
|  | 21-24 | 715 | 63.7 |
|  | > 24 | 263 | 23.4 |
| **3. Education level** | 1^st^/2^nd^/3^rd^ | 524 | 46.7 |
|  | 4^th^/5^th^/Master’s | 598 | 53.3 |
| **4. Current living area** | Urban | 727 | 64.8 |
|  | Rural | 395 | 35.2 |
| **5. Monthly family income (BDT)** | < 25,000 | 436 | 38.9 |
|  | 25,000 to 50,000 | 478 | 42.6 |
|  | > 50,000 | 208 | 18.5 |
| **6. Total online educational duration per day** | 0 to below 2 h | 214 | 19.1 |
|  | 2 to below 4 h | 370 | 33.0 |
|  | 4 to below 6 h | 366 | 32.6 |
|  | ≥ 6 h | 172 | 15.3 |
| **7. University type** | Private | 619 | 55.2 |
|  | Public | 367 | 32.7 |
|  | Others | 136 | 12.1 |
| **Psychological distress** | No distress (well) | 253 | 22.5 |
|  | Mild | 105 | 9.4 |
|  | Moderate | 94 | 8.4 |
|  | Severe | 670 | 59.7 |
